# Mechanistic Insights into the Role of Artificial Intelligence and Machine Learning in the Diagnosis and Management of Multiple Sclerosis

**DOI:** 10.3390/pathophysiology33020035

**Published:** 2026-05-27

**Authors:** Alireza Minagar, Mohammadali Sahraian

**Affiliations:** 1 Multiple Sclerosis Research Center, Neuroscience Institute, Sina Hospital, Hassan Abad Square, Imam Khomeini Street, Tehran 1136746911, Iran; msahrai@tums.ac.ir; 2 School of Cybersecurity and Information Technology, University of Maryland Global Campus, Adelphi, MD 20783, USA

**Keywords:** multiple sclerosis, artificial intelligence, machine learning, MRI segmentation, SuStaIn, MindGlide, neurofilament light chain, disease-modifying therapy, drug discovery, digital biomarkers, mechanistic interpretability, BTK inhibitors, federated learning

## Abstract

Multiple sclerosis (MS) is a chronic, immune-mediated demyelinating disease of the central nervous system whose heterogeneous clinical, radiological, and biological course has long resisted precise individual-level prediction. The recent convergence of large longitudinal datasets, advanced computational methods, and increasingly informative biomarkers has created conditions in which artificial intelligence (AI) and machine learning (ML) can begin to address that problem substantively. This review surveys the current evidence for AI/ML applications across the MS care continuum, with particular focus on the literature from 2022 through early 2026. Nine domains are examined: automated MRI lesion segmentation and quantification, fluid biomarker interpretation, unsupervised disease subtyping, disability progression prediction, treatment response stratification, drug repurposing and molecular discovery, digital biomarker monitoring, mechanistic interpretability, and integrated clinical management protocols. Notable recent contributions include the SuStaIn-based identification of two biologically distinct MS trajectories distinguished by early versus late serum neurofilament light chain elevation, the MindGlide deep learning platform enabling longitudinal analysis of archived routine clinical MRI data, the T-cell morphological classifier predicting natalizumab treatment response before drug initiation, and the fenebrutinib Phase III program that produced the first Bruton’s tyrosine kinase inhibitor results meeting primary endpoints in both relapsing and primary progressive MS. A proposed AI-Enhanced Management Protocol (AMP-26) reflecting 2026 clinical standards is included as an appendix. Throughout, emphasis is placed on mechanistic interpretability: the distinction between models that correlate features with outcomes and models whose decision logic reflects established MS pathobiology is considered a prerequisite for clinical credibility and regulatory readiness.

## 1. Introduction

For many years multiple sclerosis (MS) has been defined by a paradox. Some patients with the same diagnosis have courses that vary widely despite apparently similar onset: similar burden of lesions on MRI, similar relapse history, and similar response to initial treatment that do not predict dramatically different disability outcomes over the course of the next decade. This variability was once considered noise in the data, but it is now clear that these distinctions represent the biological hallmarks of distinct mechanisms driving the disease at variable pace and in differing combinations within each patient (recently confirmed with AI-based models in *Nature Medicine* and *Brain* showing ongoing subclinical inflammation and continued accrual of disease burden even during periods of clinical quiescence) [1,2]. MS is a disease that affects nearly 2.9 million people globally and is the most common non-traumatic cause of neurologic disability among young adults [3]. As our treatment options have grown (currently there are over twenty MS disease-modifying therapies [DMTs] ranging from platform injectables to high-efficacy B-cell, integrin, and sphingosine-1-phosphate receptor-targeting monoclonal antibodies [mAbs]) [4], our desire to get these treatment decisions right for each person has intensified. Choosing the right DMT to start, for the right patient, and at the right time, has never been more important in MS. Guidelines developed from population-level data leave much to be desired when treating our individual patients.

Artificial intelligence and machine learning represent the first analytical frameworks with both the dimensionality tolerance and the pattern-recognition capacity to address this problem at the individual level. AI/ML methods can integrate high-dimensional, multimodal, longitudinal data streams—MRI lesion maps, retinal nerve fiber measurements, serum neurofilament concentrations, genomic risk profiles, wearable sensor outputs, and electronic health records—and extract from that heterogeneous mass a mechanistic portrait of the individual patient’s disease state and trajectory [5]. The vision of AI-driven personalized care across the full MS continuum has been formally articulated in the clinical literature, encompassing disease monitoring, treatment response stratification, and biomarker-guided therapeutic decision-making as interdependent components of a unified precision medicine framework [6].

Standardized clinical disability assessment is itself a structured data stream amenable to ML analysis. Greselin and colleagues recently demonstrated this in a machine learning analysis of 13,103 Neurostatus-eEDSS assessments from the EXPAND trial in secondary progressive MS, identifying four distinct disability subscore patterns within identical EDSS scores at and above 4.0—biological heterogeneity invisible to the composite score but recoverable through ML applied to the underlying examination structure [7]. Genetic susceptibility variables (HLA-DRB1*15:01 carrier status, polygenic risk scores) and environmental exposure history (Epstein–Barr virus serology, vitamin D status, smoking history) constitute additional structured input variables that further enrich AI/ML model specifications when consortium datasets permit their inclusion.

This review surveys the current state of evidence across the full AI/ML application space in MS, from lesion detection and biomarker interpretation through drug discovery and integrated clinical protocols. The emphasis throughout is on mechanism: not merely predictive accuracy, but the extent to which AI model outputs connect to established MS pathobiology in ways that generate scientifically credible hypotheses and support defensible clinical decisions [8].

Three points of emphasis distinguish the present review from prior surveys of AI/ML in MS [4,5,6]. First, every methodological development discussed below is mapped back to the three pathophysiological pillars established in Section 2—peripheral immune-mediated inflammation, demyelination, and axonal degeneration—rather than presented as an isolated computational achievement. Second, the review prioritizes mechanistic interpretability over predictive accuracy as the criterion for clinical readiness, on the grounds that a model whose decision logic does not connect to established MS biology cannot be expected to generalize beyond its training distribution and cannot be defended to a patient or to a regulatory body. Third, the integrated clinical management protocol presented in Appendix A (AMP-26) is offered as a conceptual framework illustrating how the reviewed advances might converge in clinical practice, with the explicit understanding that prospective validation of every decision rule remains the precondition for actual deployment.

## 2. Pathophysiology of Multiple Sclerosis: A Framework for AI/ML Applications

Three mechanisms are central to MS pathogenesis: peripheral infiltration of immune cells resulting in CNS inflammation mediated by autoreactive CD4+ helper T cells, CD8+ cytotoxic T cells, and B lymphocytes that breach the blood–brain barrier; destruction of myelin sheaths by infiltrating immune cells and activated microglia; and axonal damage resulting from chronic inflammation and loss of myelin-derived trophic support [8,9]. The rate of disease progression and severity is influenced by additional factors, including the HLA-DRB1*15:01 susceptibility allele, prior Epstein–Barr virus infection, vitamin D insufficiency, and age-related decline in CNS remyelination capacity [8]. Combined with the underlying inflammatory and neurodegenerative pathology, these factors contribute to the clinical variability of MS.

Recently, the concept of Progression Independent of Relapse Activity (PIRA) has been formally recognized and defined. PIRA refers to the accumulation of irreversible disability between and independent of clinical relapses, driven by smoldering intrathecal inflammation and chronic neurodegeneration that operate below conventional monitoring thresholds [10]. In a pooled analysis of two major randomized controlled trials, Kappos and colleagues demonstrated that PIRA accounts for the majority of confirmed disability accumulation even in typical relapsing-remitting MS—a finding that fundamentally challenges the historical assumption that relapse suppression is sufficient to prevent disability accumulation [11]. PIRA constitutes both a mechanistic insight and a clinical imperative: it demands monitoring tools capable of detecting subclinical disease activity, precisely the domain in which AI/ML approaches can offer the most meaningful clinical contribution.

The growing centrality of PIRA to MS clinical research has made operational harmonization of its definition a methodological priority. Müller et al. addressed this in a 2023 systematic review of 119 published studies, identifying substantial variability in baseline anchoring, confirmation interval, and relapse-free window definitions, and proposing a unified PIRA definition combining a roving baseline, a 24-week confirmation interval, and a defined relapse-free interval surrounding the disability event [12]. The implications extend beyond trial design to AI/ML methodology directly: models trained on inconsistently labeled disability progression learn the labeling inconsistency as much as the underlying biology, and predictions degrade systematically when transferred to centers using different operational PIRA definitions.

Each pathophysiological process corresponds to measurable biomarkers that serve as candidate inputs and target variables for AI/ML models. Inflammatory activity is quantified by gadolinium-enhancing lesions on MRI and by CSF (cerebrospinal fluid) markers including oligoclonal bands, CXCL13, and interleukin-6. Demyelination is reflected in magnetization transfer ratio and myelin water fraction. Neurodegeneration manifests as brain atrophy, retinal nerve fiber layer (RNFL) thinning on optical coherence tomography (OCT), and serum neurofilament light chain (sNfL) concentration—a structural protein released from injured axons, measurable in peripheral blood through ultrasensitive single-molecule array (Simoa) assays [8,13]. A mechanistically grounded AI/ML model for MS maps these biomarkers to the biological processes they represent rather than treating them as opaque statistical predictors. This distinction is of fundamental importance for model interpretability, clinical credibility, and the generation of testable mechanistic hypotheses.

## 3. Data Sources and Preprocessing for MS-Focused AI/ML Models

The data landscape for MS AI/ML research encompasses a progressively widening array of modalities. Structural MRI provides information on lesion burden, lesion distribution, white matter integrity, and regional brain atrophy with high spatial resolution, connecting directly to inflammatory and demyelinating disease mechanisms [14]. OCT quantification of peripapillary RNFL thickness and ganglion cell–inner plexiform layer (GCIP) volume provides a non-invasive surrogate for cumulative axonal loss, reflecting neurodegenerative processes that structural MRI may underestimate [15]. CSF biomarkers—oligoclonal bands, cytokine panels, albumin index, and neurofilament proteins—characterize immunological and structural CNS compartment states that peripheral blood measurements incompletely capture [13]. Genomic data inform heritable disease susceptibility and pharmacogenomic variation. Clinical disability indices, including the Expanded Disability Status Scale (EDSS), timed functional tests, and patient-reported outcome measures, have well-recognized limitations in sensitivity and scope that constrain their capacity to capture the full burden of disease impact and subclinical progression independent of relapses [16]. Wearable accelerometers and gyroscopes extend monitoring to continuous real-world conditions, capturing gait, physical activity, and circadian behavioral patterns.

Susceptibility-sensitive sequences extend the structural MRI feature set with paramagnetic rim lesions (PRLs), chronic active lesions identifiable by an iron-laden microglial rim at the lesion edge that signals smoldering compartmentalized inflammation persisting after acute demyelination has resolved. The North American Imaging in Multiple Sclerosis Cooperative consensus statement from Bagnato et al. established standardized radiological criteria for PRL identification and recognized PRL burden as an imaging biomarker of chronic active disease, with mechanistic relevance to PIRA that complements conventional T2 lesion metrics [17]. Automated PRL detection has itself become an active AI/ML application: Lou and colleagues demonstrated fully automated PRL identification on 3T susceptibility-based MRI by integrating T2*-magnitude and unwrapped phase contrasts in a method designed to scale to large clinical cohorts where manual rim assessment is infeasible [18]. Inclusion of PRL counts in multimodal AI/ML pipelines is therefore directly relevant to the same compartmentalized inflammatory mechanism that drives PIRA, providing a structural correlate of the biology that fluid biomarkers detect downstream.

Each modality presents distinct preprocessing requirements that must be addressed before AI/ML models can be validly applied. MRI data require bias field correction, skull stripping, and spatial normalization; center-specific acquisition protocols introduce systematic variance that harmonization pipelines must address without suppressing genuine biological signal. The importance of anticipating deployment conditions during model development rather than treating generalization as a secondary concern has been articulated by Behar and colleagues as a fundamental principle of scalable medical AI [19]. Longitudinal MS datasets present particular challenges: irregular visit intervals, non-random missing data patterns, and the prognostic information embedded in the spacing between observations all require methodological attention beyond standard imputation approaches. Pinto and colleagues demonstrated that multimodal integration—combining MRI volumetrics, sNfL, and OCT—consistently outperforms single-modality models in MS prediction tasks, reflecting the complementary mechanistic information provided by imaging, fluid, and functional biomarker streams [20].

**Figure 1:**
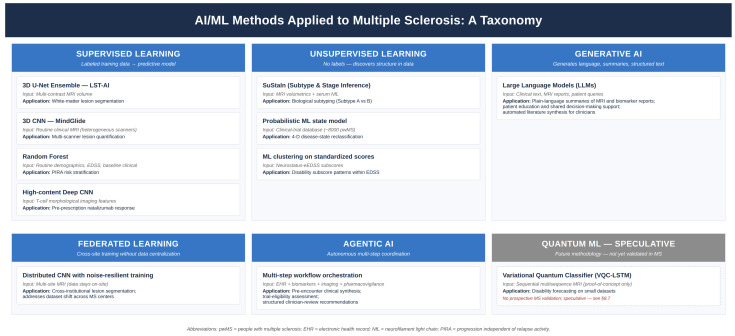
Taxonomy of artificial intelligence and machine learning methods applied to multiple sclerosis, organized by learning paradigm. Each panel groups representative methods cited in this review by their methodological category, with the corresponding input data type and intended clinical application. Supervised learning: 3D U-Net Ensemble (LST-AI) for white-matter lesion segmentation [21]; 3D convolutional neural network (MindGlide) for multi-scanner lesion quantification on routine clinical MRI [22]; Random Forest for progression-independent of relapse activity (PIRA) risk stratification from routine demographics and baseline clinical variables [23]; high-content deep convolutional neural network for pre-prescription prediction of natalizumab response from T-cell morphological imaging features [24]. Unsupervised learning: SuStaIn (Subtype and Stage Inference) for biological subtyping from MRI volumetrics and serum neurofilament light chain (NfL) [2]; probabilistic machine-learning state model for four-dimensional disease-state reclassification from a clinical-trial database of approximately 8000 people with multiple sclerosis (pwMS) [1]; machine-learning clustering on standardized Neurostatus-eEDSS subscores for the identification of disability subscore patterns within the Expanded Disability Status Scale [7]. Generative AI: Large language models for plain-language summaries of MRI and biomarker reports, patient education and shared decision-making support, and automated literature synthesis for clinicians [25,26]. Federated learning: distributed convolutional neural network with noise-resilient training for cross-institutional lesion segmentation, addressing dataset shift across MS centers without data centralization [27]. Agentic AI: multi-step workflow orchestration integrating electronic health record (EHR) data, biomarkers, imaging, and pharmacovigilance inputs for pre-encounter clinical synthesis, trial-eligibility assessment, and structured clinician-review recommendations [28]. Quantum machine learning (speculative): Variational Quantum Classifier (VQC-LSTM) for disability forecasting on small datasets [29]; no prospective MS validation has been reported, and this category is presented as future methodology rather than current clinical capability (see Section 8.7). Color coding: panels with blue category headers denote AI/ML methodologies with established or actively developing applications in MS research; the panel with a gray header and red label (Quantum Machine Learning) is included as a speculative methodological frontier without prospective MS validation. Abbreviations: pwMS = people with multiple sclerosis; EHR = electronic health record; NfL = neurofilament light chain; PIRA = progression independent of relapse activity; EDSS = Expanded Disability Status Scale.

The validity of any AI/ML model in MS depends critically on the validity of its training labels, and clinical disability scoring imposes specific quality requirements that distinguish it from imaging or biomarker inputs. EDSS scoring is operator-dependent and subject to inter-rater variance that, when uncorrected, propagates into ML models as label noise—a model trained on noisy labels learns the noise rather than the underlying clinical signal. Standardization through the certified Neurostatus protocol and its electronic counterpart (Neurostatus-eEDSS), which enforces structured Functional System scoring with automated consistency checks and requires rater certification through documented training, reduces this label noise to a level at which subsequent ML analysis can recover biologically meaningful structure rather than rater idiosyncrasy. The Greselin study cited in the Introduction provides direct evidence that high-quality, standardized scoring enables ML to extract clinically relevant subgroup heterogeneity that the composite EDSS conceals by design [7]. Figure 1 summarizes the AI/ML methodological taxonomy referenced throughout this review.

## 4. Diagnostic Applications: Imaging, Biomarkers, and Multimodal Approaches

### 4.1. Automated MRI Lesion Segmentation

Automated white-matter lesion segmentation represents the most clinically mature AI application in MS. Manual segmentation by expert neuroradiologists is accurate but operator-dependent, poorly scalable, and subject to inter-rater variability that compromises longitudinal monitoring. Deep learning architectures derived from the encoder–decoder U-Net family—employing successive convolutional layers with skip connections that preserve fine spatial detail during upsampling—now achieve segmentation performance at or near expert neuroradiologist level on established benchmarks [30].

**Figure 2:**
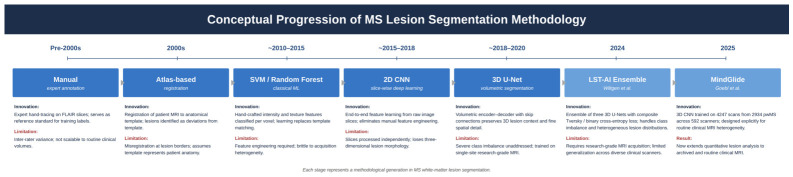
Conceptual progression of multiple sclerosis (MS) white-matter lesion segmentation methodology, from manual expert annotation through current deep-learning ensembles capable of operating on routine clinical MRI. Each stage represents a methodological generation, with its central innovation and the principal limitation it addresses shown below the corresponding box. **Darker blue boxes denote historical methodological generations (Pre-2000s through ~2018–2020); lighter blue boxes denote contemporary state-of-the-art methods (2024 onwards).** Pre-2000s: manual expert hand-tracing on FLAIR slices, which serves as the reference standard for training labels but suffers from inter-rater variance and limited scalability. 2000s: atlas-based registration of patient MRI to an anatomical template, identifying lesions as deviations from the template; misregistration at lesion borders is a key failure mode. ~2010–2015: classical machine learning with hand-crafted intensity and texture features classified per voxel using support vector machines (SVM) or random forests, replacing template matching with learning at the cost of feature-engineering effort and acquisition-heterogeneity brittleness. ~2015–2018: 2D convolutional neural networks (CNNs) introduce end-to-end feature learning from raw image slices but process slices independently, losing three-dimensional lesion morphology. ~2018–2020: 3D U-Net architectures with skip connections preserve volumetric lesion context and fine spatial detail [30]; severe class imbalance and reliance on single-site research-grade MRI remain unresolved. 2024: the LST-AI ensemble combines three 3D U-Nets with a composite Tversky and binary cross-entropy loss to address class imbalance and heterogeneous lesion distributions, though acquisition still requires research-grade MRI [21]. 2025: MindGlide, a 3D CNN trained on 4247 scans from 2934 people with multiple sclerosis (pwMS) across 592 scanners, is designed explicitly for routine clinical MRI heterogeneity and extends quantitative lesion analysis to archived clinical scans [22]. Abbreviations: MS = multiple sclerosis; pwMS = people with multiple sclerosis; MRI = magnetic resonance imaging; FLAIR = fluid-attenuated inversion recovery; SVM = support vector machine; CNN = convolutional neural network.

Two recent contributions merit specific attention. Ashtari and colleagues developed a pre-activation U-Net for simultaneous lesion segmentation and detection, achieving an F1-score of 48.1% on a new-lesion detection benchmark—a meaningful advance over classical automated methods [30]. Wiltgen and colleagues subsequently introduced LST-AI (Lesion Segmentation Tool-AI): an open-source ensemble of three 3D U-Nets trained on 491 annotated MRI pairs from people with MS (pwMS), employing a composite Tversky/binary cross-entropy loss function specifically designed to address the severe class imbalance between lesioned and non-lesioned white matter voxels. LST-AI demonstrated substantially superior performance to its predecessor on heterogeneous lesion distributions encountered in routine clinical practice [21]. Real-world validation is equally important: Barnett and colleagues confirmed across multiple Australian MS centers that AI-based MRI monitoring translates to meaningful reductions in radiologist reporting time without loss of clinical accuracy [31], and Peters and colleagues reported concordant findings in a European institutional setting [32]. Figure 2 illustrates the conceptual progression of MS lesion segmentation methodology from manual annotation through MindGlide.

### 4.2. MindGlide: Repurposing Clinical MRI Archives

Most automated segmentation tools require standardized, high-resolution MRI acquisitions. This constraint limits their utility for the large volumes of routine clinical imaging that have accumulated in hospital picture archiving and communication systems over years—data that could provide valuable longitudinal disease tracking information if computationally accessible. Goebl, Wingrove, and colleagues—under the supervision of Eshaghi at UCL—addressed this limitation with MindGlide, a 3D CNN (convolutional neural network) trained on 4247 brain MRI scans from 2934 pwMS across 592 scanners, specifically designed to accommodate the acquisition heterogeneity of routine clinical practice, including variable field strengths (1.5 T to 3 T), variable slice thickness, motion artifacts, and diverse MRI pulse sequences [22]. MindGlide processes a standard clinical scan in under 10 s and demonstrates approximately 60% improvement in lesion localization relative to SAMSEG, the prior reference tool, while also outperforming WMH-SynthSeg. The clinical significance is substantial: millions of previously unanalyzable archived clinical MRI scans become accessible for retrospective longitudinal analysis, and every prospectively acquired routine clinical scan becomes immediately compatible with quantitative treatment monitoring without requiring research-grade acquisition protocols [22].

#### Architectural Basis of MindGlide: 3D CNN Lesion Detection

The architectural choice of three-dimensional convolution is mechanistically appropriate for MS lesion detection. Standard two-dimensional CNNs process individual MRI slices independently, losing the volumetric continuity essential for characterizing lesion geometry, periventricular distribution patterns, and the juxtacortical or infratentorial anatomical locations that carry diagnostic weight under the 2024 McDonald Criteria [22]. MindGlide’s 3D CNN applies convolutional filters simultaneously across all three spatial dimensions, enabling detection of lesion morphology—including the ovoid periventricular orientation characteristic of MS and the cortical surface proximity of juxtacortical lesions—that no individual axial slice can fully represent.

The encoder component of the architecture applies successive 3D convolutions with ReLU (rectified linear unit) activations and max-pooling to progressively compress spatial resolution while building hierarchical feature representations, from low-level edge detection to higher-order recognition of T2 hyperintensity patterns and central vein geometry. The decoder employs transposed convolutions to restore spatial resolution, with skip connections from corresponding encoder layers preserving fine structural detail suppressed during downsampling—the design principle shared with the LST-AI ensemble and the broader family of U-Net-derived segmentation architectures [22,30].

### 4.3. AI-Driven Disease Subtyping: The SuStaIn Framework

Perhaps the most conceptually significant recent contribution to MS diagnostics is not an incremental improvement in lesion detection but a fundamental revision of disease classification. Willard and colleagues, working under the supervision of Eshaghi at UCL, applied the Subtype and Stage Inference (SuStaIn) unsupervised machine learning framework—previously applied to MS subtyping using single-visit MRI data alone [33]—to a combined dataset integrating MRI volumetrics with serum neurofilament light chain measurements. Training on 189 patients with RRMS and SPMS with validation in 445 newly diagnosed patients, the study—published in *Brain* in December 2025—identified two biologically distinct MS trajectories that cut across conventional clinical phenotype boundaries [2], representing a data-driven reclassification with direct therapeutic implications.

Subtype A, designated the Early-sNfL subtype, is characterized by elevated sNfL concentrations in the early disease course concurrent with accelerated lesion development in the corpus callosum, indicating an inflammation-dominant biological profile with active axonal injury occurring before significant brain atrophy is measurable by conventional volumetry. Patients classified to Subtype A carry a meaningfully higher risk of rapid disability accumulation when treated with standard first-line injectable DMTs, establishing a mechanistic rationale for immediate high-efficacy intervention—specifically, B-cell depleting agents such as ocrelizumab or ofatumumab—independent of presenting EDSS score or relapse frequency [2].

Subtype B, the Late-sNfL subtype, presents a clinically distinct challenge: progressive atrophy of limbic cortex and deep grey matter structures precedes any significant elevation in blood neurofilament concentrations. The neurodegeneration is biologically active and structurally consequential, yet it does not produce the inflammatory biomarker signal by which conventional monitoring systems detect disease activity [2]. By the time sNfL becomes elevated in Subtype B patients, irreversible structural damage has accrued. The clinical priority for this group is neuroprotection and intensive grey-matter surveillance rather than escalation of anti-inflammatory therapy—a fundamentally different management strategy from Subtype A that population-level treatment guidelines cannot encode. Prior MRI-only applications of SuStaIn had already established that data-driven subtypes predict long-term disability outcomes more accurately than clinical phenotype labels [33], and the addition of sNfL to the subtyping framework deepens the biological specificity of these distinctions substantially.

### 4.4. Biomarker-Driven Diagnosis and Prognostication

Serum neurofilament light chain has emerged as the most versatile blood-based biomarker in MS, reflecting axonal injury across all phenotypes and disease stages. Its clinical value depends critically, however, on the analytical framework through which individual measurements are interpreted. Single-timepoint thresholds referenced against age-stratified population norms have limited sensitivity to within-individual change. Benkert and colleagues addressed this by developing a personalized sNfL reference model, validated retrospectively in large observational cohorts, that generates individualized prognostic estimates by accounting for age, body mass index, and the patient’s own longitudinal trajectory—enabling detection of clinically meaningful sNfL elevation at the individual level rather than requiring population-threshold exceedance [34].

Meier and colleagues subsequently demonstrated in a JAMA Neurology study that serum GFAP (glial fibrillary acidic protein)—a marker of astrocyte activation and CNS structural damage—provides independent prognostic information beyond sNfL, particularly for the progressive disability accumulation that characterizes secondary progressive MS and that sNfL alone does not fully capture [35]. The practical implication is that a combined AI-interpreted biomarker panel, with model outputs accounting for longitudinal trajectories in both sNfL and GFAP, would outperform either marker interpreted in isolation. This is a computational task that AI systems can perform in real time at the point of care, and one for which no reliable manual equivalent exists.

Targeted prediction of PIRA has received dedicated methodological attention in the recent literature. Betti and colleagues, in a multicenter Italian cohort study of 719 newly diagnosed pwMS, applied multiple machine learning models to predict relapse-independent disability progression within the first three years of diagnosis using routine clinical and demographic data [23]. The best-performing Random Forest classifier achieved an area under the ROC curve of 0.75, rising to 0.77 in the subgroup of patients under 45 years of age. The most predictive features were EDSS at 24 months, age at symptom onset, and disease duration at baseline—variables available in routine clinical practice—indicating that ML-based PIRA risk stratification does not require advanced imaging or biomarker data to generate clinically meaningful predictions.

## 5. Prognostication and Disease Course Prediction

The diagnostic and biomarker applications surveyed in Section 4 establish individual-level disease characterization at the time of presentation. The clinical question that follows is what will happen next: which pwMS will progress, on what timescale, and through which mechanism. This question maps to all three pathophysiological pillars simultaneously—inflammatory disease activity drives near-term relapse risk, demyelination and remyelination determine functional recovery between relapses, and axonal degeneration drives long-term disability accumulation including PIRA. Predictive models that disentangle the three contributions, rather than collapsing them into a single composite outcome score, generate clinical decisions that are mechanistically defensible rather than merely accurate.

Machine learning methods have been applied to the prediction of first clinical relapse, time to conversion from clinically isolated syndrome (CIS) to definite MS [36,37], and long-term disability progression following DMT initiation. Performance has improved progressively as training datasets have grown and architectural choices have become more sophisticated. The central unresolved question is no longer whether ML prediction outperforms unaided clinical judgment—the evidence supporting modest but meaningful predictive superiority has accumulated sufficiently. The question is whether that superiority is robust enough across varied deployment conditions, and clinically actionable enough in prospective settings, to justify integration into routine decision-making workflows.

The most rigorously designed evaluation of this question to date was conducted by De Brouwer and colleagues using MSBase consortium data spanning 146 MS centers across 40 countries—15,240 patients and more than 283,000 longitudinal clinical episodes—to train and evaluate multiple ML architectures for two-year disability progression prediction [38]. By permutation importance analysis, the most influential predictive variable was the EDSS score at the time of prediction, followed by the mean EDSS trajectory over the preceding three years. Model calibration was satisfactory. The authors concluded nonetheless that the predictive performance achieved had not reached the level of clinical trust necessary for routine adoption—an assessment that accurately reflects the gap between research demonstration and validated clinical tool, and that the field has not yet systematically closed.

A conceptually distinct approach was published in 2025 by Ganjgahi, Häring and colleagues in Nature Medicine. Applying probabilistic machine learning to a clinical trial database comprising approximately 8000 patients, 118,000 patient visits, and more than 35,000 MRI scans from the Novartis-Oxford MS dataset, the authors proposed abandoning categorical clinical phenotyping entirely in favor of a four-dimensional disease state representation capturing physical disability, brain damage, relapse activity, and subclinical radiological activity [1]. Transitions between states were modeled with quantified probabilities, and DMT effects were estimated at the level of individual state transitions—demonstrating that treatment benefits differ meaningfully by disease-state trajectory rather than by phenotype label alone. This reclassification has direct implications for adaptive trial design, treatment escalation criteria, and the interpretation of DMT comparative effectiveness data.

Transcriptomic prognostication represents a complementary approach with particular relevance for primary progressive MS, where conventional monitoring tools have demonstrated limited sensitivity. Published in Brain Communications in 2025, a study employing ML analysis of the peripheral blood transcriptome in people with primary progressive MS (pwPPMS) identified a 10-gene expression signature predictive of brain volume loss and disability progression over a 120-week observation window [39]. If validated prospectively in independent cohorts, this approach would enable prognostic stratification of pwPPMS from a routine blood draw before MRI-detectable structural change accrues, opening an intervention window that does not currently exist in clinical practice.

Additional modeling contributions include a deep learning approach by Storelli and colleagues that predicted MS disease progression from longitudinal MRI sequences by extracting subtle volumetric changes across sequential scans without reliance on clinical disability scoring [40], and an explainable ML framework by Campanioni and colleagues applying baseline MRI features to simultaneous prediction of multiple MS trajectory descriptors, with SHAP (SHapley Additive exPlanations)-derived feature attributions that aligned with established MS pathological anatomy [41].

## 6. Therapeutic Decision Support and Management Optimization

### 6.1. Individual Treatment Response Prediction

Prognostication establishes which pwMS need treatment intensification; therapeutic decision support establishes which treatment best matches the underlying biology of the individual patient. The mechanistic asymmetry of currently available DMTs—uniformly anti-inflammatory at the periphery, with limited capacity to address demyelination or compartmentalized intrathecal neurodegeneration—means that AI-guided treatment selection should explicitly model which of the three pathophysiological pillars dominates the individual patient’s disease state, and should match drug mechanism to that biology rather than to a population-level escalation algorithm.

Treatment selection in MS has historically followed a population-level escalation logic in which patients initiate lower-efficacy therapy and advance to higher-efficacy agents upon evidence of breakthrough disease activity. This paradigm carries both clinical and safety costs: the patient may accumulate irreversible disability during ineffective treatment, and high-efficacy DMTs such as natalizumab carry serious adverse risks—including progressive multifocal leukoencephalopathy mediated by JC virus reactivation—that make prescribing them to patients unlikely to benefit an unfavorable risk-benefit calculation. Multi-omics classifiers combining MRI radiomics, sNfL, and immune cell phenotyping have demonstrated the capacity to identify natalizumab non-responders with greater than 80% probability before treatment initiation, as reviewed by Amin and colleagues [4].

The most compelling recent advance in this area was published by Chaves and colleagues in *Nature Communications* in 2025. Their platform applies high-content automated imaging to T-cell morphology in vitro, extracting more than 400 features characterizing the shape, protein localization, and motility of CD8+ T cells from patient peripheral blood samples, and trains a deep learning classifier on those morphological profiles to predict natalizumab treatment response at the individual patient level before drug exposure [24]. Across an independent multicenter validation cohort, the classifier achieved approximately 92% accuracy in predicting individual treatment response. This performance, if replicated in prospective clinical deployments, would represent a patient-specific pre-prescription stratification tool sufficient to materially change the clinical risk-benefit calculation for natalizumab initiation.

### 6.2. Treatment Monitoring and Adverse Event Prediction

Longitudinal sNfL monitoring as a treatment response biomarker has received formal consensus endorsement. The eBioMedicine CMSC Consensus guidance published in 2024 recommends sNfL monitoring alongside MRI and clinical assessment, with AI-assisted personalized reference modeling for individual-level interpretation [42]. Data from the NaloMS cohort, incorporated into that consensus, demonstrated that persistently elevated sNfL despite DMT initiation predicted relapse-free disability progression and conversion to secondary progressive MS with 82% accuracy in multivariable modeling. The clinical operationalization of this finding—automated sNfL trend surveillance with triggered reassessment at a pre-specified elevation threshold—is technically feasible within existing electronic health record infrastructure.

Adverse event prediction represents an underexplored application of the same monitoring infrastructure. JC virus antibody index trajectories, lymphocyte count kinetics during sphingosine-1-phosphate receptor modulator therapy, and hepatic enzyme trends during BTK inhibitor exposure are structured longitudinal data streams amenable to ML-based surveillance at detection sensitivities exceeding intermittent clinical review [43,44]. Integration of adverse event prediction into the same AI monitoring layer as treatment response surveillance requires no additional data infrastructure and represents a straightforward near-term implementation target.

## 7. Artificial Intelligence in Drug Discovery and Repurposing

### 7.1. The Unmet Therapeutic Need: Remyelination and Neuroprotection

Where prognostication identifies who is at risk and therapeutic decision support optimizes use of currently available agents, drug discovery addresses the limits of the therapeutic armamentarium itself—specifically, the persistent gap between the inflammation-targeted DMTs that exist and the remyelination-promoting and neurodegeneration-arresting agents that the pathophysiology demands. The current MS pharmacological landscape, despite its breadth, is characterized by a fundamental therapeutic asymmetry: every approved DMT targets peripheral immune mechanisms, and none has demonstrated reliable capacity to promote remyelination of chronically demyelinated axons or arrest the smoldering intrathecal neurodegeneration responsible for PIRA [45,46]. This gap defines the most important domain for AI-assisted drug discovery in MS—one requiring identification of targets in oligodendrocyte biology, remyelination signaling pathways, and microglial activation, and the development of CNS-penetrant small molecules acting on mechanisms that current immunosuppressive DMTs do not address.

### 7.2. Network Medicine, Graph Neural Networks, and Target Identification

The network medicine framework proposes that therapeutic targets are most likely to succeed when they occupy biological network positions proximal to disease-associated nodes—a principle with empirical support demonstrated by Ruiz and colleagues using a multiscale interactome approach, wherein drugs acting in network neighborhoods close to disease-associated proteins showed superior clinical efficacy to those acting at greater topological distance [47]. Graph neural networks (GNNs), which operate natively on graph-structured protein interaction and pathway databases, are well-suited to implementing this framework for MS-specific target identification.

In application to MS, Liu and colleagues employed the Drug-Gene Interaction Database (DGIdb) to integrate transcriptomic and proteomic data from MS tissue studies, identifying eculizumab—a complement inhibitor currently approved for paroxysmal nocturnal hemoglobinuria—as a candidate drug targeting complement receptor 1 (CR1), a gene implicated in MS immune regulation and identified in MS genome-wide association studies [48]. Li and colleagues extended the computational drug repurposing framework with their DTD-GNN model, which simultaneously models drug-target-disease ternary relationships in heterogeneous knowledge graphs, surfacing candidates whose predicted binding profiles align with disease-relevant network topology [49].

### 7.3. Virtual Cell Platforms and AI-Accelerated Screening (2026)

Virtual cell platforms—AI-driven computational simulations of disease-affected cellular environments—enable evaluation of novel drug-cell interaction profiles before any physical synthesis or wet-laboratory assay, compressing early-stage screening timelines substantially and allowing only the highest-probability candidates to advance to resource-intensive laboratory validation [50].

### 7.4. Translating Remyelination Therapeutics into Clinical Practice: Drug Candidates Identified Through AI-Assisted Discovery

Green et al. provided the proof-of-principle demonstration of pharmacological remyelination in humans. In a randomized, double-blind, placebo-controlled trial (known as ReBUILD), clemastine fumarate improved visual evoked potential latency in pwMS with chronic demyelinating optic neuropathy [51]. Clemastine is an antihistamine drug that was repurposed because of its antimuscarinic effects. Clemastine was identified through phenotypic screening of chemical libraries. Presently, the capacity exists to interrogate libraries of compounds orders of magnitude larger than we could ever realistically screen in vitro through the application of AI-based virtual screening algorithms. Nakamura et al. demonstrated that ibudilast, a phosphodiesterase inhibitor with inherent anti-neuroinflammatory and neuroprotective properties, decreased the burden of slowly enlarging lesions in progressive MS [52]. Slowly enlarging lesions serve as a radiological signature of progressive disease which can be used as a proxy for ongoing intrathecal inflammation and have been shown to carry direct implications for the efficacy of PIRA-suppressive therapies.

### 7.5. Patient-Derived Organoids and AI-Powered Drug Screens as a Preclinical Standard of Care (2026)

Clayton and colleagues at the New York Stem Cell Foundation derived iPSC (induced pluripotent stem cell) lines from individuals with all three principal MS subtypes: relapsing-remitting, primary progressive, and secondary progressive. After differentiating these lines into glia-enriched cultures, the group applied single-cell transcriptomic profiling to characterize glia-intrinsic disease mechanisms [53]. Two findings stood out. Cultures derived from primary progressive MS donors showed reduced oligodendrocyte counts, and both oligodendrocyte lineage cells and astrocytes exhibited upregulation of immune and inflammatory gene expression. Because these signatures arose in cultures free of peripheral immune cells, the abnormalities cannot be attributed to circulating lymphocytes; this finding argues that pwMS-derived iPSC models can serve as a substrate for identifying glia-specific therapeutic targets.

### 7.6. AI-Optimized Clinical Trial Design and Pharmacovigilance

Drug discovery requires clinical validation, and AI is beginning to improve the efficiency of MS clinical trials at multiple stages. Predictive enrichment algorithms identify patient populations with disease activity profiles sufficient to detect treatment effects at specified sample sizes, reducing required enrollment and shortening trial timelines—a methodology reviewed in the context of autoimmune drug development by Moingeon and colleagues [54]. The disease-state reclassification framework of Ganjgahi and colleagues [1] creates a data-driven basis for stratified enrollment in mechanism-specific trials: rather than enrolling by phenotype label, future BTK inhibitor or remyelination trials could enrich for patients in disease states where the drug’s mechanism of action is biologically most relevant. Natural language processing applied to pharmacovigilance adverse event narratives provides an additional AI contribution to post-market drug safety monitoring, identifying signals in unstructured text that escape structured adverse event coding [55].

## 8. Future Directions: The 2026 Predictive Frontier

### 8.1. Proteomic Aging and Pre-Diagnostic Biomarker Detection

Hamitouche and colleagues at McGill University presented at the 2026 ACTRIMS Forum preliminary results from a study applying ML to thousands of proteins in blood and brain tissue from UK Biobank participants who subsequently developed MS, reporting proteomic aging signatures detectable up to 11 years before the first clinical diagnosis [56]. In the abstract, the authors reported preliminary findings suggesting that the brains of pwMS may be biologically aged on the order of 2.5 years beyond their chronological age as measured by proteomic composition; these data have not yet been published in peer-reviewed form, and prospective validation in independent longitudinal biobank cohorts will be required before any clinical inference can be drawn. Should the pre-diagnostic window suggested by these preliminary findings be confirmed in peer-reviewed prospective work, AI-driven proteomic surveillance of at-risk populations—defined by family history, genetic susceptibility, or prior Epstein–Barr virus serostatus—could in principle identify candidates for primary neuroprotection before clinical MS manifests.

### 8.2. Digital Biomarkers and Wearable Sensor Monitoring

Reliance on annual or biannual MRI and clinic-based disability assessment for MS monitoring creates systematic detection gaps during which subclinical neurodegeneration proceeds undetected. Fitzgerald and colleagues at Johns Hopkins School of Medicine addressed this limitation in a study published on 4 March 2026 in *Neurology*, demonstrating that wrist-worn accelerometer sensors monitoring daily physical activity in a cohort of 238 MS patients over approximately three years identified individuals at higher risk of disability worsening and brain volume loss [57]. A within-person decrease in physical activity specifically between 8:00 and 10:00 a.m. was associated with a 20% higher risk of confirmed disability worsening, and each standard deviation reduction in morning activity was associated with 0.34% greater loss of deep grey matter volume and 0.35% greater reduction in thalamic volume. This finding suggests that passively collected wearable sensor data, analyzed by AI in continuous background surveillance, could generate an early warning signal for subclinical progression actionable in the interval between scheduled clinic assessments.

The broader vision of continuous digital monitoring is a real-time AI layer operating alongside routine clinical care, generating longitudinal behavioral and physiological records that complement episodic clinical assessments and imaging. Realizing this vision requires integration of wearable data streams with biomarker monitoring and imaging data in a unified AI-managed patient model—the digital twin concept whose technical infrastructure is being actively developed.

### 8.3. Agentic AI in Clinical Workflow Integration

By mid-2026, agentic AI frameworks—autonomous systems capable of coordinating complex multi-step workflows without discrete human prompting—had begun to demonstrate practical utility in MS research and clinical environments. In contrast to reactive AI systems that generate outputs in response to specific queries, agentic systems can autonomously integrate electronic health records, genomic data, longitudinal imaging, real-time biomarker feeds, and pharmacovigilance databases to generate clinical trial eligibility assessments, flag adverse event signals, and update disease trajectory predictions [28]. In one illustrative paradigm, prior to a patient encounter, such a system autonomously reviews updated sNfL results, identifies relevant imaging changes, cross-references findings against the PIRA risk model, and prepares a structured evidence-based treatment recommendation for clinician review. The clinician’s role in this workflow is evaluative rather than computational, preserving human judgment while substantially reducing the time and cognitive load required for data synthesis.

### 8.4. Mechanistic Interpretability, Trustworthy AI, and Regulatory Considerations

The 2024 EU AI Act mandates explainability for AI tools designated as medical devices, requiring documentation of the features and reasoning contributing to model outputs—a standard that both clinicians and patients require to engage meaningfully with AI-generated recommendations [58]. In MS, this requirement is scientifically as well as regulatorily important: a model achieving high predictive accuracy through exploitation of scanner-site-specific imaging artifacts, demographic proxies, or center-level prescribing patterns will degrade upon deployment at new centers and may generate false mechanistic inferences that misdirect subsequent research. SHAP (SHapley Additive exPlanations) values provide post hoc feature attribution for tabular clinical models; gradient-weighted class activation maps (Grad-CAM) serve the analogous function for imaging models. When attribution outputs align with known MS pathological anatomy—periventricular lesion load, thalamic atrophy, optic nerve involvement—such correspondence constitutes convergent evidence that the model has learned biologically meaningful representations rather than confounding statistical regularities.

Human–machine hybrid decision architectures, in which clinician judgment is combined with ML model output, represent the appropriate model for near-term clinical AI deployment: not autonomous AI-driven decision-making, but AI-augmented clinical reasoning in which the model extends the clinician’s capacity to synthesize multidimensional longitudinal data while human expertise provides the contextual judgment and accountability that AI systems cannot substitute. This framework is consistent with the evidence base for clinical decision support across multiple domains of medicine and has been endorsed by regulatory guidance for high-risk AI systems in healthcare settings.

The regulatory pathway for clinical deployment of AI/ML tools in MS warrants more specific articulation than the general EU AI Act framing above provides. Tools such as MindGlide and SuStaIn-MRI fall within the diagnostic and prognostic Software as a Medical Device (SaMD) classification under both the EU Medical Device Regulation and the FDA SaMD framework; their evidence requirements differ in important respects from those for treatment-decision support tools. For diagnostic and prognostic SaMD, prospective multicenter validation against reference standards in clinically representative populations is generally the expected evidence package, rather than randomized controlled trial demonstration of patient outcome benefit, on the basis that the tool informs a clinical decision rather than directing it. Tools such as the Chaves T-cell morphological classifier [24], by contrast, propose to direct treatment selection and would require evidence of patient outcome benefit—plausibly through randomized comparison of AI-stratified versus standard-of-care natalizumab initiation. Post-deployment governance of AI/ML medical devices is structurally different from that of fixed-formulation drugs because the underlying models can be updated with new training data and architectural changes that materially alter performance. The FDA Predetermined Change Control Plan framework and the analogous provisions emerging under the EU AI Act allow for governed model updating within pre-specified bounds, with re-validation requirements scaled to the magnitude of change; both frameworks place explicit obligations on the deploying institution to monitor model performance against held-out validation data continuously and to detect performance degradation arising from dataset shift before clinical errors accumulate. The practical implication for MS AI research is that any tool intended for actual clinical deployment must be designed from initial development with a defined regulatory pathway, a pre-specified evidence package, and a continuous performance monitoring infrastructure, rather than as a research demonstration retrofitted with regulatory documentation after the fact.

### 8.5. Outstanding Mechanistic Research Priorities

Several fundamental mechanistic questions relevant to MS pathobiology remain inadequately addressed by existing AI/ML research and constitute high-priority targets for future investigation. The relative contribution of peripheral inflammation versus compartmentalized intrathecal neurodegeneration to PIRA has not been quantified with sufficient precision across MS subtypes, age groups, and disease durations to guide individualized treatment allocation decisions. Whether demyelination and inflammation represent truly independent pathophysiological processes, or whether one drives the other through specific molecular intermediaries detectable in longitudinal multi-omics data, has not been resolved [8,56]. The mechanistic role of Epstein–Barr virus reactivation in relapse precipitation, and the feasibility of detecting premonitory immune signatures in blood or CSF that are amenable to AI-based classification, remains an open question of considerable therapeutic relevance. Answering these questions will require consortium-style datasets linking registries such as MSBase and NARCOMS with imaging archives, biobanks, and pharmacovigilance databases. The framework for enabling this kind of collaborative infrastructure in dementia research has been proposed by Ranson and colleagues and is directly applicable to MS [59].

### 8.6. BTK Inhibitors and the Role of AI in Mechanism-Stratified Trial Design

Bruton’s tyrosine kinase (BTK) inhibitors represent the first MS therapeutic class pharmacologically designed to target CNS-resident immune processes as well as peripheral inflammation—BTK being expressed by both B lymphocytes and microglia, the latter constituting the primary cellular driver of the compartmentalized intrathecal inflammation underlying PIRA. The 2025–2026 Phase III trial results in this class are both clinically significant and mechanistically instructive, as reviewed comprehensively by Naydovich and colleagues [60].

Fenebrutinib (Genentech/Roche), a reversible, non-covalent BTK inhibitor engineered for high CNS penetrance and 130-fold selectivity for BTK over closely related kinases, achieved the first positive Phase III results for any BTK inhibitor in both relapsing and primary progressive MS. The FENhance 1 and 2 trials demonstrated statistically significant reductions in annualized relapse rate versus teriflunomide over at least 96 weeks of treatment, and the FENtrepid trial in PPMS demonstrated non-inferiority to ocrelizumab—the only approved PPMS therapy—in delaying composite confirmed disability progression over 120 weeks, with a consistent numerical advantage over ocrelizumab apparent from week 24; complete datasets from these studies were submitted for regulatory review in 2026 [60].

The broader BTK inhibitor evidence base reveals mechanistically important differential outcomes. Tolebrutinib (Sanofi) achieved a 31% reduction in confirmed disability progression risk in the HERCULES trial for non-relapsing secondary progressive MS—a population for which no approved therapy previously existed [61]—but failed to achieve primary endpoint reductions in annualized relapse rate in the GEMINI 1 and 2 relapsing MS trials [62]. In December 2025, tolebrutinib also failed to meet its primary endpoint in the PERSEUS trial for primary progressive MS; peer-reviewed publication of these results is pending [60]. Evobrutinib (Merck KGaA) failed both Phase III EvolutionRMS trials in relapsing MS and was discontinued from further development [60,63].

The aim of the discussion that follows is forward-looking and methodological rather than retrospective and causal: we propose how the AI-derived stratification tools described earlier in this section could be applied to the design of future BTK inhibitor trials, not how patient selection might have explained the outcomes of trials already conducted. The negative trial results reviewed above admit multiple non-exclusive mechanistic interpretations—differential CNS penetrance across compounds, varying degrees of peripheral B-cell modulation, dose and exposure differences across study designs, and stratification of enrolled populations—that cannot be adjudicated from currently available data. The differential performance of these agents is mechanistically interpretable and directly relevant to AI-guided patient stratification. Favorable outcomes in progressive and non-relapsing populations are consistent with meaningful CNS penetration and microglial targeting; failure in relapsing populations at the same nominal target may reflect insufficient peripheral B-cell modulation, differential CNS penetrance across compounds, or patient selection confounds. Prospective identification of the patient population most likely to benefit from CNS-penetrant BTK inhibitors—characterized by AI-derived disease-state classification [1] combined with SuStaIn biological subtyping [2], sNfL trajectory modeling [34], and PIRA risk assessment [23]—represents one of the most operationally feasible and clinically consequential near-term applications of MS AI research. The analytical tools for this stratification exist; their application to BTK inhibitor trial enrollment criteria has not yet been attempted prospectively.

### 8.7. Quantum Machine Learning: A Speculative Methodological Frontier

Quantum machine learning (QML) is included here as a forward-looking methodological development whose application to MS remains at an early proof-of-concept stage rather than as an established analytical tool. By applying quantum computational principles to high-dimensional pattern recognition, QML may eventually offer capacity to process multi-omics datasets at scales that classical algorithms cannot support; the published MS-related work to date, however, has been confined to small proof-of-concept datasets without independent validation, and no prospective evaluation in MS cohorts of clinically relevant size has been reported [29]. The methodology is included in this review for completeness, with the explicit caveat that current evidence does not support its present use in clinical or translational MS research; its eventual relevance to MS is contingent on technical maturation of quantum computational infrastructure and on prospective validation in independent MS cohorts rather than on demonstrated current utility.

## 9. Validation, Generalizability, and Clinical Integration Challenges

### 9.1. Generalizability, Dataset Shift, and Federated Learning

The preponderance of published AI/ML research in MS is retrospective. Models are typically trained on historical data from academic MS centers with systematic data collection practices, evaluated on held-out subsets of the same data distribution, and reported with performance metrics that reflect internal validity but provide limited assurance about generalizability to new clinical environments. Dataset shift—the systematic divergence between training and deployment data distributions arising from differences in MRI acquisition protocols, patient demographics, diagnostic criteria applied across institutions or time periods, and local DMT prescribing norms—is the primary mechanism by which research demonstrations fail to translate into functional clinical tools. Behar and colleagues have characterized this as a fundamental challenge requiring explicit methodological attention during the design phase of medical AI development [19], a position corroborated by the practical failure modes documented by Kernbach and Staartjes in the context of ML-based clinical prediction modeling broadly [64].

The international MSBase disability progression study by De Brouwer and colleagues, the most methodologically comprehensive published attempt to construct a generalizable MS prediction model, enrolled data from 146 centers across 40 countries and achieved promising calibration and discrimination. The authors’ conclusion that predictive performance had not yet reached clinical-trust threshold for routine adoption reflects an accurate and intellectually honest assessment of where the field stands [38]. Federated learning—training models across distributed hospital networks without centralizing patient-level data—addresses the dataset shift and data governance problems simultaneously by enabling model training on the full distribution of clinical practice variability rather than on a single-center or multi-center convenience sample. Bai and colleagues demonstrated the technical feasibility of federated learning for MS lesion segmentation across heterogeneous clinical sites, with performance approximating centralized training [27].

### 9.2. Regulatory Framework and Algorithmic Equity

The EU AI Act’s conformity assessment requirements for software as a medical device (SaMD) constitute an appropriate regulatory framework for AI-driven MS clinical tools, requiring pre-market evidence of data quality, risk mitigation strategies, and post-market performance surveillance before deployment [58]. These requirements are demanding, but they reflect the genuine clinical stakes involved in deploying probabilistic decision-support systems in conditions where model errors have direct consequences for patient care. Algorithmic equity represents a parallel obligation. The majority of large MS AI training datasets derive from European and North American academic referral centers; the resulting models encode assumptions about imaging acquisition standards, genotypic background, and healthcare access patterns that may not generalize to African, Asian, Latin American, or under-resourced clinical populations with distinct MS epidemiology. Sendak and colleagues have argued that systematic equity auditing—evaluation of model performance across demographic subgroups—should be a required component of any clinical AI validation study, not an optional supplement to primary efficacy assessment [65].

### 9.3. AI in Patient Communication, Health Literacy, and Shared Decision-Making

The therapeutic and prognostic complexity described throughout this review creates a substantive communication challenge: the information that neurologists must now synthesize to make individualized treatment decisions—incorporating sNfL trajectories, biological subtypes, MRI volumetrics, pharmacogenomic profiles, and probabilistic outcome models—exceeds what most patients can access or interpret without structured support. Effective shared decision-making requires that patients have meaningful access to the reasoning behind clinical recommendations, a requirement that existing communication channels do not reliably fulfill for MS decisions of this complexity.

Large language model (LLM)-based tools are beginning to address this gap substantively. Ziemssen and colleagues documented in *Multiple Sclerosis Journal* that adapted LLMs can outperform medical experts on clinical text summarization tasks—condensing complex MRI reports, biomarker assessments, and treatment decision rationales into plain-language summaries accessible to patients with varied health literacy [25]. Inojosa and colleagues demonstrated that ChatGPT (GPT-4, OpenAI, San Francisco, CA, USA)could explain MS concepts at a patient-accessible level with clinically acceptable accuracy while demonstrating appropriate attribution of the hallucination risk and knowledge currency limitations that require physician oversight [26].

Helme and colleagues provided an important patient-perspective complement to these technical findings in a 2025 *Frontiers in Immunology* study examining patient values, priorities, and concerns regarding AI use in MS management [66]. Patients with MS expressed strong preference for AI tools that increase transparency—specifically, tools capable of explaining not merely the predictive output but the biological features driving it and their implications for individual treatment options. This preference aligns precisely with the mechanistic interpretability requirements imposed by the EU AI Act and articulated by the clinical AI research community; it establishes that explainability is a patient-centered requirement, not solely a regulatory or scientific one, and strengthens the ethical case for prioritizing interpretable model architectures in MS clinical AI development.

Algorithms cannot substitute for PROMs or for things patients live with. Any defensible clinical AI/ML workflow must involve both. What gets fed into AI pipelines—imaging volumes, fluid biomarkers, EDSS scores—is only part of what MS actually does to patients. Fatigue, cognitive symptoms, lost work capacity, mood changes, and Quality-of-life decline: These are what patients actually experience. And the standard inputs grossly underestimate them. The clinical heterogeneity that AI models attempt to characterize biologically is paralleled by heterogeneity in how individual pwMS experience and prioritize their own disease. Two patients with identical sNfL trajectories and identical SuStaIn subtype assignments may diverge sharply in what each considers an acceptable disability trajectory, an acceptable adverse event risk, or an acceptable monitoring intensity—and that divergence reflects not noise but legitimately different value structures that cannot be derived from the underlying biology. ML pipelines that incorporate PROMs as both input variables and outcome targets are technically feasible (the EXPAND-derived ML analysis of Greselin and colleagues [7] is structurally compatible with PROM augmentation) and are increasingly recognized as a research priority within the MS clinical AI community. Shared decision-making in this framework treats AI output as one input to clinical reasoning rather than as a recommendation to be ratified: the model provides a structured probability estimate; the clinician contextualizes that estimate against the full picture of clinical examination, comorbidity, and prognostic biomarker burden; and the patient supplies the values and priorities that determine which probability-weighted outcome is preferable. This three-way exchange is the operational meaning of patient-centered AI in MS, and it is the standard against which any deployed AI/ML decision-support tool should be evaluated.

Practical LLM applications in MS clinical communication currently in development include automated generation of personalized post-visit summaries translating clinical decisions into plain language, patient-facing intelligent portals responding to inquiries about sNfL values, MRI findings, and DMT adverse effect profiles using MS-specific fine-tuned language models, and NLP-driven medication adherence monitoring systems detecting symptom pattern changes indicative of emerging relapse or tolerability problems before scheduled clinic assessment [25,66]. The same LLM infrastructure supports research administration and clinician education, enabling real-time synthesis of rapidly expanding MS literature, automated generation of grant abstracts and regulatory documentation, and point-of-care literature integration. As reviewed in the context of neurology education by Figari Jordan and colleagues in 2024, these capabilities represent foundational infrastructure for translating AI research discoveries into accessible clinical practice rather than peripheral conveniences [67].

## 10. Comparative Analysis of Leading AI Frameworks in MS (2025)

Table 1 provides a structured comparison of MindGlide and SuStaIn across eight analytical dimensions, illustrating how imaging automation and biological subtyping function as complementary tools within an integrated AI-enhanced diagnostic workflow rather than as competing methodological alternatives [2,22].

The Human–AI Hybrid Diagnostic Workflow (2026)

The integrated use of MindGlide and SuStaIn in sequence constitutes a three-phase AI-augmented diagnostic protocol. In Phase 1, MindGlide is applied to the initial diagnostic MRI to identify and quantify white matter lesions, brain atrophy, and treatment effects, flagging cases meeting high-efficacy intervention criteria based on imaging biology alone, independent of presenting EDSS score [22]. In Phase 2, SuStaIn integrates the MindGlide imaging output with baseline sNfL to determine biological subtype, producing the mechanistic phenotype classification that drives treatment stratification [2]. In Phase 3, the SuStaIn subtype classification informs DMT selection: Subtype A (Early-sNfL) patients are directed to immediate high-efficacy B-cell depletion therapy, bypassing the step-therapy escalation sequence; Subtype B (Late-sNfL) patients receive neuroprotective and grey-matter-focused management. At each phase, the output is presented to the treating neurologist as structured evidence supporting—but not replacing—the clinical decision.

## 11. Conclusions

AI and ML have moved from early promise to incipient clinical utility within the last few years. Automated MS lesion segmentation on MRI using deep learning algorithms has reached expert neuroradiologist-level performance at mass-analysis scales impossible for human reading. Unsupervised ML enabled the discovery of biologically defined MS subtypes (based on whether regional sNfL elevations precede or follow localized brain atrophy) that clinically defined MS phenotypes do not capture, with implications for initial DMT choice and better prediction of response to treatment. AI-guided longitudinal assessment of fluid biomarkers, especially paired sNfL and GFAP levels, facilitates disease prognosis at an individual level that population-threshold methodologies cannot achieve. Imaging neuroimmune phenotypes based on morphological profiling of blood T-cells can predict response to natalizumab treatment before prescription at the individual patient level. Generative AI for drug discovery is accelerating the timeline for discovery and preclinical validation of remyelination and neuroprotective therapies. The successful Phase III trials of fenebrutinib in 2025 marked the first effective translation of a BTK inhibitor for MS and moved the treatment mechanism into both relapsing disease and PPMS.

Acknowledging this tremendous growth in AI for MS requires recognition of outstanding challenges the field has yet to overcome. Many contributions of AI/ML to MS research are retrospective analyses that have not yet been validated prospectively on independent clinical cohorts. Dataset shift, AI bias, and the EU AI Act substantially increase the bar of evidence needed for AI to be used in clinical practice, a bar that many published models fail to reach. Mechanistic interpretability, while crucial for enabling effective AI integration into clinical decision-making, is inconsistently practiced and rarely prioritized for validation. Existing ML applications in MS have not consistently demonstrated fair generalization across diverse demographic groups.

AI’s most significant impact on our understanding of multiple sclerosis may be a reframing of the disease concept itself. By elucidating that MS exists on a biological gradient rather than within siloed clinical categories, and that specific, biologically distinguishable processes inform individualized disease courses years before patients show clinical signs of progression, AI has laid the groundwork for precision medicine in MS that treating patients by population-wide algorithms cannot achieve. Ensuring that these new tools are prospectively validated, interpretable, and available equitably to all patients is the next hurdle for the field.

## Figures and Tables

**Table 1:** Comparative analysis of MindGlide (imaging automation) and SuStaIn (biological subtyping) as leading AI diagnostic frameworks in MS, 2025. CVS: Central Vein Sign; sNfL: serum neurofilament light chain; SAMSEG: Sequence Adaptive Multimodal SEGmentation; WMH-SynthSeg: White Matter Hyperintensity SynthSeg; DMT: disease-modifying therapy.

Feature	MindGlide (Imaging Automation)	SuStaIn (Biological Subtyping)
**Developer/Origin**	University College London (UCL)/Queen Square Institute of Neurology (2025)	UCL Centre for Medical Image Computing—Willard et al., 2025—*Brain* 148(12):4578 [2]
**Primary Input**	Routine clinical MRI scans (any single contrast: T2-weighted, FLAIR, T1)	Multi-modal: MRI volumetrics + Serum Neurofilament Light Chain (sNfL)
**AI Architecture**	3D Convolutional Neural Networks (3D CNNs)—trained on 4247 scans from 592 scanners	Unsupervised Machine Learning—Subtype and Stage Inference (SuStaIn) algorithm
**Mechanistic Focus**	Automated detection and quantification: white matter lesion volume, brain atrophy, treatment effects	Temporal modeling: sequence of sNfL elevation relative to regional MRI atrophy patterns
**Key Output**	Lesion volume and brain tissue metrics from archival single-contrast scans; treatment effect detection	Classification into Early-sNfL (Subtype A) or Late-sNfL (Subtype B) with staging
**Clinical Advantage**	Unlocks archived routine MRI data; 60% improvement over SAMSEG; 5–10 s per scan	Predicts disability trajectory and treatment response years before clinical deterioration
**Impact on Management**	Real-world treatment monitoring; retrospective longitudinal analysis without research-grade acquisitions	Precision DMT selection by biological subtype: B-cell depletion (Subtype A) vs. neuroprotection (Subtype B)
**Representative Reference**	Goebl et al., 2025—*Nat Commun* 16:3149 [22]	Willard et al., 2025—*Brain* 148(12):4578 [2]

## Data Availability

No new data were created or analyzed in this review. Data sharing is not applicable to this article.

## Appendix A. 2026 AI-Enhanced Multiple Sclerosis Management Protocol (AMP-26)

The AMP-26 protocol represents a proposed, evidence-informed management framework synthesized from published literature through March 2026. It has not been prospectively validated, and the specific decision rules and alert criteria proposed below are illustrative only, drawn from the cited research literature where applicable but not validated as decision thresholds in prospective clinical use. AMP-26 is presented as a conceptual integration to illustrate how current AI/ML advances might be operationalized in clinical practice; any actual clinical deployment would require prospective validation of each decision rule and threshold in a manner equivalent to that required for software as a medical device under the EU AI Act and analogous regulatory frameworks. The protocol operationalizes the diagnostic and therapeutic developments reviewed above into a structured clinical management framework. Its defining departure from conventional practice is the initiation of multimodal AI analysis at the time of first neurological presentation—Radiologically Isolated Syndrome (RIS) or Clinically Isolated Syndrome (CIS)—rather than requiring confirmation of a second relapse before biological characterization and treatment stratification are undertaken.

### Appendix A.1. Phase I: First-Contact Diagnostic Suite

**Step 1: Automated Lesion Profiling via MindGlide**

Immediate AI-based analysis of the initial diagnostic MRI to quantify white matter lesion volume and brain tissue metrics [22]. **Decision rule:** Evidence of active lesion accrual or significant brain volume loss at presentation triggers High-Efficacy Early Intervention classification independent of current EDSS score. Imaging-defined biological urgency supersedes clinical disability as the primary treatment allocation criterion.

**Step 2: Biological Subtyping via SuStaIn**

**Subtype A (Early-sNfL):** Elevated sNfL concurrent with corpus callosum lesion accrual. Inflammation-dominant biological profile. Immediate B-cell depletion therapy recommended. Conventional step-therapy escalation bypassed on the basis of biological subtype classification [2].

**Subtype B (Late-sNfL):** Progressive limbic cortex and deep grey matter atrophy preceding sNfL elevation. Neurodegeneration-dominant biological profile. Early neuroprotective agents and intensive grey-matter surveillance protocol recommended [2].

### Appendix A.2. Phase II: Dynamic Monitoring via Digital Twin

Rather than relying on episodic clinic assessments and scheduled annual MRI, the AMP-26 monitoring framework maintains a continuously updated Digital Twin for each patient—a computational model integrating all available longitudinal data to provide ongoing disease trajectory estimation.

**Passive Wearable Monitoring**

Continuous wrist-worn accelerometer monitoring of daily physical activity patterns [57]. **Alert threshold:** A clinically meaningful sustained reduction in morning activity, with thresholds to be established prospectively in validation cohorts, triggers an unscheduled sNfL measurement to assess for subclinical neurodegeneration preceding clinical detection.

**Predictive PIRA Screening**

Six-monthly recalculation of individual PIRA risk using the validated Random Forest classifier [23]. High-risk classification triggers formal treatment review and consideration of CNS-penetrant high-efficacy DMT escalation or enrollment in an eligible remyelination clinical trial.

### Appendix A.3. Phase III: AI-Assisted Therapeutic Decision Support

**T-Cell Morphological Pre-Prescription Assessment**

Prior to natalizumab initiation, T-cell morphological profiling is performed via the Chaves deep learning classifier [24]. A predicted non-responder classification, with the operational probability cut-point to be established and validated prospectively, triggers recommendation to explore alternative DMTs, avoiding unnecessary exposure to PML risk in pwMS with low predicted therapeutic benefit.

**Transcriptomic Progression Monitoring in PPMS**

Annual assessment of the 10-gene peripheral blood transcriptomic signature in primary progressive MS patients [39]. Signature indicating high neurodegeneration risk triggers pre-emptive treatment intensification before structural deterioration is measurable by standard MRI volumetry.

### Appendix A.4. AMP-26 Protocol Summary

**Table A1:** Comparison of pre-2024 conventional MS management targets against 2026 AI-driven targets under the AMP-26 protocol. EDSS: Expanded Disability Status Scale; NEDA-3: No Evidence of Disease Activity (relapses, disability progression, new MRI lesions); PIRA: Progression Independent of Relapse Activity; DMT: disease-modifying therapy; sNfL: serum neurofilament light chain.

Metric	Traditional Goal (Pre-2024)	2026 AI-Driven Target (AMP-26)
**Diagnosis Speed**	Months to years after second clinical relapse	Days via automated lesion profiling at first neurological event
**Progression Tracking**	EDSS score at clinic visit every 6–12 months	Continuous digital biomarkers + sNfL kinetics in real time
**Treatment Strategy**	Escalation: initiate low-efficacy therapy, escalate on failure	Induction approach: subtype-specific high-efficacy therapy from diagnosis
**Success Definition**	NEDA-3 (No Evidence of Disease Activity)	PIRA-Zero: absence of silent neurodegeneration confirmed by digital monitoring
**Drug Selection**	Empirical or guideline-based escalation	T-cell morphological profiling to predict individual DMT response before prescribing
**Patient Monitoring**	Annual or biannual MRI	Wearable-based passive monitoring with AI-driven anomaly detection

## References

[1] Ganjgahi H., Häring D.A., Aarden P., Graham G., Sun Y., Gardiner S., Su W., Berge C., Bischof A., Fisher E. (2025). AI-driven reclassification of multiple sclerosis progression. Nat. Med..

[2] Willard C., Puglisi L., Ravi D., Dmitrieva M., Mattiesing R.M., Barkhof F., Alexander D.C., Harlow D.E., Piani-Meier D., Eshaghi A. (2025). Combined magnetic resonance imaging and serum analysis reveals distinct multiple sclerosis types. Brain.

[3] Lublin F.D., Reingold S.C., Cohen J.A., Cutter G.R., Sørensen P.S., Thompson A.J., Wolinsky J.S., Balcer L.J., Banwell B., Barkhof F. (2014). Defining the clinical course of multiple sclerosis: The 2013 revisions. Neurology.

[4] Amin M., Martínez-Heras E., Ontaneda D., Prados Carrasco F. (2024). Artificial intelligence and multiple sclerosis. Curr. Neurol. Neurosci. Rep..

[5] Yousef H., Malagurski Tortei B., Castiglione F. (2024). Predicting multiple sclerosis disease progression and outcomes with machine learning and MRI-based biomarkers: A review. J. Neurol..

[6] Praet J., Anderhalten L., Comi G., Horakova D., Ziemssen T., Vermersch P., Lukas C., van Leemput K., Steppe M., Aguilera C. (2024). A future of AI-driven personalized care for people with multiple sclerosis. Front. Immunol..

[7] Greselin M., Lu P.J., Mroczek M., Cerdá-Fuertes N., Demirtzoglou A., Papadopoulou A., Kuhle J., Leppert D., Arnould S., Aoun M. (2025). AI-assisted identification of disability patterns within identical EDSS grades. Mult. Scler..

[8] Reich D.S., Lucchinetti C.F., Calabresi P.A. (2018). Multiple Sclerosis. N. Engl. J. Med..

[9] Thompson A.J., Banwell B.L., Barkhof F., Carroll W.M., Coetzee T., Comi G., Correale J., Fazekas F., Filippi M., Freedman M.S. (2018). Diagnosis of multiple sclerosis: 2017 revisions of the McDonald criteria. Lancet Neurol..

[10] Ciccarelli O., Barkhof F., Calabrese M., De Stefano N., Eshaghi A., Filippi M., Gasperini C., Granziera C., Kappos L., Rocca M.A. (2024). Using the progression independent of relapse activity framework to unveil the pathobiological foundations of multiple sclerosis. Neurology.

[11] Kappos L., Wolinsky J.S., Giovannoni G., Arnold D.L., Wang Q., Bernasconi C., Model F., Koendgen H., Manfrini M., Belachew S. (2020). Contribution of Relapse-Independent Progression vs Relapse-Associated Worsening to Overall Confirmed Disability Accumulation in Typical Relapsing Multiple Sclerosis in a Pooled Analysis of 2 Randomized Clinical Trials. JAMA Neurol..

[12] Müller J., Cagol A., Lorscheider J., Tsagkas C., Benkert P., Yaldizli Ö., Kuhle J., Derfuss T., Sormani M.P., Thompson A. (2023). Harmonizing definitions for progression independent of relapse activity in multiple sclerosis: A systematic review. JAMA Neurol..

[13] Kontopodis E.E., Papadaki E., Trivizakis E., Maris T.G., Simos P., Papadakis G.Z., Tsatsakis A., Spandidos D.A., Karantanas A., Marias K. (2021). Emerging deep learning techniques using magnetic resonance imaging data applied in multiple sclerosis and clinical isolated syndrome patients (Review). Exp. Ther. Med..

[14] Rathmann E., Hemkemeier P., Raths S., Grothe M., Mankertz F., Hosten N., Flessa S. (2024). Changes in MRI workflow of multiple sclerosis after introduction of an AI-software: A qualitative study. Healthcare.

[15] Dongil-Moreno F.J., Ortiz M., Pueyo A., Boquete L., Sánchez-Morla E.M., Jimeno-Huete D., Miguel J.M., Barea R., Vilades E., Garcia-Martin E. (2024). Diagnosis of multiple sclerosis using optical coherence tomography supported by explainable artificial intelligence. Eye.

[16] Fuchs T.A., Schoonheim M.M., Zivadinov R., Dwyer M.G., Colato E., Weinstock Z., Weinstock-Guttman B., Strijbis E.M., Benedict R.H. (2024). Cognitive progression independent of relapse in multiple sclerosis. Mult. Scler..

[17] Bagnato F., Sati P., Hemond C.C., Elliott C., Gauthier S.A., Harrison D.M., Mainero C., Oh J., Pitt D., Shinohara R.T. (2024). Imaging chronic active lesions in multiple sclerosis: A consensus statement. Brain.

[18] Lou C., Sati P., Absinta M., Clark K., Dworkin J.D., Valcarcel A.M., Schindler M.K., Reich D.S., Sweeney E.M., Shinohara R.T. (2021). Fully automated detection of paramagnetic rims in multiple sclerosis lesions on 3T susceptibility-based MR imaging. Neuroimage Clin..

[19] Zvuloni E., Celi L.A., Behar J.A. (2023). Generalization in medical AI: A perspective on developing scalable models. arXiv.

[20] Pinto M.F., Oliveira H., Batista S., Cruz L., Pinto M., Correia I., Martins P., Teixeira C. (2020). Prediction of disease progression and outcomes in multiple sclerosis with machine learning. Sci. Rep..

[21] Wiltgen T., McGinnis J., Schlaeger S., Kofler F., Voon C., Berthele A., Bischl D., Grundl L., Will N., Metz M. (2024). LST-AI: A deep learning ensemble for accurate MS lesion segmentation. Neuroimage Clin..

[22] Goebl P., Wingrove J., Abdelmannan O., Brito Vega B., Stutters J., Ramos S.D.G., Kenway O., Rossor T., Wassmer E., Arnold D.L. (2025). Enabling new insights from old scans by repurposing clinical MRI archives for multiple sclerosis research. Nat. Commun..

[23] Poretto V., Endrizzi W., Betti M., Bovo S., Bellinvia A., Ragni F., Lapucci C., Moroni M., Marangoni S., Portaccio E. (2025). Machine learning analysis applied to prediction of early progression independent of relapse activity in multiple sclerosis patients. Eur. J. Neurol..

[24] Chaves B., Santos E Silva J.C., Nakaya H., Socquet-Juglard N., Bucciarelli F., Prunier G., Almeida M.V., Lacouture C., Kari S., Astier A.L. (2025). In vitro morphological profiling of T cells predicts clinical response to natalizumab therapy in patients with multiple sclerosis. Nat. Commun..

[25] Inojosa H., Voigt I., Wenk J., Ferber D., Wiest I., Antweiler D., Weicken E., Gilbert S., Kather J.N., Akgün K. (2024). Integrating large language models in care, research, and education in multiple sclerosis management. Mult. Scler..

[26] Inojosa H., Gilbert S., Kather J.N., Proschmann U., Akgün K., Ziemssen T. (2023). Can ChatGPT explain it? Use of artificial intelligence in multiple sclerosis communication. Neurol. Res. Pract..

[27] Bai L., Wang D., Wang H., Barnett M., Cabezas M., Cai W., Calamante F., Kyle K., Liu D., Ly L. (2024). Improving multiple sclerosis lesion segmentation across clinical sites: A federated learning approach with noise-resilient training. Artif. Intell. Med..

[28] Hinostroza Fuentes V.G., Karim H.A., Tan M.J.T., AlDahoul N. (2025). AI with agency: A vision for adaptive, efficient, and ethical healthcare. Front. Digit. Health.

[29] Mayfield J., El Naqa I. (2024). Evaluation of VQC-LSTM for disability forecasting in multiple sclerosis using sequential multisequence MRI. Quantum Mach. Intell..

[30] Ashtari P., Barile B., Van Huffel S., Sappey-Marinier D. (2022). New multiple sclerosis lesion segmentation and detection using pre-activation U-Net. Front. Neurosci..

[31] Barnett M., Wang D., Beadnall H., Bischof A., Brunacci D., Butzkueven H., Brown J.W.L., Cabezas M., Das T., Dugal T. (2023). A real-world clinical validation for AI-based MRI monitoring in multiple sclerosis. npj Digit. Med..

[32] Peters S., Kellermann G., Watkinson J., Gärtner F., Huhndorf M., Stürner K., Jansen O., Larsen N. (2024). AI supported detection of cerebral multiple sclerosis lesions decreases radiologic reporting times. Eur. J. Radiol..

[33] Pontillo G., Penna S., Cocozza S., Quarantelli M., Gravina M., Lanzillo R., Marrone S., Costabile T., Inglese M., Brescia Morra V. (2022). Stratification of multiple sclerosis patients using unsupervised machine learning: A single-visit MRI-driven approach. Eur. Radiol..

[34] Benkert P., Meier S., Schaedelin S., Manouchehrinia A., Yaldizli Ö., Maceski A., Oechtering J., Achtnichts L., Conen D., Derfuss T. (2022). Serum neurofilament light chain for individual prognostication of disease activity in people with multiple sclerosis: A retrospective modelling and validation study. Lancet Neurol..

[35] Meier S., Willemse E.A.J., Schaedelin S., Oechtering J., Lorscheider J., Melie-Garcia L., Cagol A., Barakovic M., Galbusera R., Subramaniam S. (2023). Serum glial fibrillary acidic protein compared with neurofilament light chain as a biomarker for disease progression in multiple sclerosis. JAMA Neurol..

[36] Zhao Y., Healy B.C., Rotstein D., Guttmann C.R., Bakshi R., Weiner H.L., Brodley C.E., Chitnis T. (2017). Exploration of machine learning techniques in predicting multiple sclerosis disease course. PLoS ONE.

[37] Zhang H., Alberts E., Pongratz V., Mühlau M., Zimmer C., Wiestler B., Eichinger P. (2019). Predicting conversion from clinically isolated syndrome to multiple sclerosis: An imaging-based machine learning approach. NeuroImage Clin..

[38] De Brouwer E., Arany A., Simm J., Moreau Y. (2024). Machine-learning-based prediction of disability progression in multiple sclerosis: An observational, international, multi-center study. PLoS Digit. Health.

[39] Gurevich M., Omer N., Zilkha-Falb R., Brill L., Rotstein D., Achiron A. (2025). Machine learning-based prediction of disease progression in primary progressive multiple sclerosis. Brain Commun..

[40] Storelli L., Azzimonti M., Gueye M., Vizzino C., Preziosa P., Tedeschi G., De Stefano N., Pantano P., Filippi M., Rocca M.A. (2022). A deep learning approach to predicting disease progression in multiple sclerosis using magnetic resonance imaging. Investig. Radiol..

[41] Campanioni S., Veiga C., Prieto-González J.M., González-Nóvoa J.A., Busto L., Martinez C., Alberte-Woodward M., García de Soto J., Pouso-Diz J., Fernández Ceballos M.L.Á. (2024). Explainable machine learning on baseline MRI predicts multiple sclerosis trajectory descriptors. PLoS ONE.

[42] Freedman M.S., Gnanapavan S., Booth R.A., Calabresi P.A., Khalil M., Kuhle J., Lycke J., Olsson T., Consortium of Multiple Sclerosis Centers (2024). Guidance for use of neurofilament light chain as a cerebrospinal fluid and blood biomarker in multiple sclerosis management. EBioMedicine.

[43] Oh J., Giacomini P.S., Yong V.W., Costello F., Blanchette F., Freedman M.S. (2024). From progression to progress: The future of multiple sclerosis. J. Cent. Nerv. Syst. Dis..

[44] Werthen-Brabants L., Dhaene T., Deschrijver D. (2025). The role of trustworthy and reliable AI for multiple sclerosis. Front. Digit. Health.

[45] Franklin R.J.M., Ffrench-Constant C. (2017). Regenerating CNS myelin—From mechanisms to experimental medicines. Nat. Rev. Neurosci..

[46] Sriwastava S., Elkhooly M., Amatya S., Shrestha K., Kagzi Y., Bhatia D., Gupta R., Jaiswal S., Lisak R.P. (2024). Recent advances in the treatment of primary and secondary progressive multiple sclerosis. J. Neuroimmunol..

[47] Ruiz C., Zitnik M., Leskovec J. (2021). Identification of disease treatment mechanisms through the multiscale interactome. Nat. Commun..

[48] Liu Y., Wang Q., Zhao Y., Liu L., Hu J., Qiao Y., Chen J., Qin C. (2024). Identification of novel drug targets for multiple sclerosis by integrating plasma genetics and proteomes. Exp. Gerontol..

[49] Li W., Ma W., Yang M., Tang X. (2024). Drug repurposing based on the DTD-GNN graph neural network: Revealing the relationships among drugs, targets and diseases. BMC Genom..

[50] Bunne C., Roohani Y., Rosen Y., Gupta A., Zhang X., Roed M., Alexandrov T., AlQuraishi M., Brennan P., Burkhardt D.B. (2024). How to build the virtual cell with artificial intelligence: Priorities and opportunities. Cell.

[51] Green A.J., Gelfand J.M., Cree B.A., Bevan C., Boscardin W.J., Mei F., Inman J., Arnow S., Devereux M., Abounasr A. (2017). Clemastine fumarate as a remyelinating therapy for multiple sclerosis (ReBUILD): A randomised, controlled, double-blind, crossover trial. Lancet.

[52] Nakamura K., Thoomukuntla B., Bena J., Cohen J.A., Fox R.J., Ontaneda D. (2024). Ibudilast reduces slowly enlarging lesions in progressive multiple sclerosis. Mult. Scler..

[53] Clayton B.L.L., Barbar L., Sapar M., Kalpana K., Rao C., Migliori B., Rusielewicz T., Paull D., Brenner K., NYSCF Global Stem Cell Array® Team (2024). Patient iPSC models reveal glia-intrinsic phenotypes in multiple sclerosis. Cell Stem Cell.

[54] Moingeon P. (2023). Artificial intelligence-driven drug development against autoimmune diseases. Trends Pharmacol. Sci..

[55] Rehman A.U., Li M., Wu B., Ali Y., Rasheed S., Shaheen S., Liu X., Luo R., Zhang J. (2024). Role of artificial intelligence in revolutionizing drug discovery. Fundam. Res..

[56] Hamitouche D., Ding Y., Rajabli R., Garcia A.M., Thebault S., Zhou S., Zimianiti I., Jacobs B.M., Falet J., Collins D. Multimodal aging signatures identify pre-diagnostic brain aging and proteomic biomarkers in MS. Proceedings of the ACTRIMS Forum.

[57] Block V.J., Cheng S., Juwono J., Cuneo R., Kirkish G., Alexander A.M., Khan M., Akula A., Caverzasi E., Papinutto N. (2026). Association of changes in activity patterns with brain atrophy and disability progression in people with multiple sclerosis. Neurology.

[58] Khan H., Alyafei K., Ullah I., Sheikh A., Zhang X., Ahmed F., Maqbool M. (2025). Integrating big data and artificial intelligence to predict progression in multiple sclerosis: Challenges and the path forward. J. Neuroeng. Rehabil..

[59] Ranson J.M., Bucholc M., Lyall D., Newby D., Winchester L., Oxtoby N.P., Veldsman M., Rittman T., Marzi S., Skene N. (2023). Harnessing the potential of machine learning and artificial intelligence for dementia research. Brain Inform..

[60] Naydovich L.R., Orthmann-Murphy J.L., Markowitz C.E. (2025). Beyond relapses: How BTK inhibitors are shaping the future of progressive MS treatment. Neurotherapeutics.

[61] Fox R.J., Bar-Or A., Traboulsee A., Oreja-Guevara C., Giovannoni G., Vermersch P., Syed S., Li Y., Vargas W.S., Turner T.J. (2025). Tolebrutinib in nonrelapsing secondary progressive multiple sclerosis. N. Engl. J. Med..

[62] Oh J., Arnold D.L., Cree B.A.C., Ionete C., Kim H.J., Sormani M.P., Syed S., Chen Y., Maxwell C.R., Benoit P. (2025). Tolebrutinib versus teriflunomide in relapsing multiple sclerosis. N. Engl. J. Med..

[63] Montalban X., Arnold D.L., Weber M.S., Staikov I., Piasecka-Stryczynska K., Willmer J., Martin E.C., Dangond F., Syed S., Wolinsky J.S. (2019). Placebo-Controlled Trial of an Oral BTK Inhibitor in Multiple Sclerosis. N. Engl. J. Med..

[64] Kernbach J.M., Staartjes V.E. (2022). Foundations of machine learning-based clinical prediction modeling: Part I—Introduction and general principles. Acta Neurochir. Suppl..

[65] Sendak M., Gao M., Nichols M., Lin A., Balu S. (2019). Machine learning in health care: A critical appraisal of challenges and opportunities. EGEMS.

[66] Helme A., Kalra D., Brichetto G., Peryer G., Vermersch P., Weiland H., White A., Zaratin P. (2025). Artificial intelligence and science of patient input: A perspective from people with multiple sclerosis. Front. Immunol..

[67] Figari Jordan R., Sandrone S., Southerland A.M. (2024). Opportunities and challenges for incorporating artificial intelligence and natural language processing in neurology education. Neurol. Educ..

